# Pediatric and Family Physicians' Attitudes Regarding Childhood Optional Vaccines During the COVID-19 Pandemic

**DOI:** 10.7759/cureus.37338

**Published:** 2023-04-09

**Authors:** Duygu Sertkaya, Semra Şen Bayturan

**Affiliations:** 1 Pediatrics, Manisa Celal Bayar University, Manisa, TUR; 2 Pediatric Infectious Diseases, Faculty of Medicine, Manisa Celal Bayar University, Manisa, TUR

**Keywords:** pediatrics, optional vaccines, family physician, manisa, türkiye, family physician (fp), pediatrician, optional vaccination, childhood

## Abstract

Introduction: To maintain high vaccination rates, vaccination interventions should be targeted according to interests such as parents' knowledge, attitudes, beliefs, and vaccine hesitancy.

Methods: This research was conducted between June 2020 and April 2021 using a questionnaire about optional vaccines (OVs) in Turkey.

Results: A total of 241 physicians participated and 14 physicians were excluded due to insufficient data. Finally, a total of 227 physicians, including 115 pediatricians and 112 family physicians, were included in the study. The mean age of pediatricians and family physicians was 33.42 ± 8.25 years and 35.46 ± 11.09 years, respectively. There was no significant difference between pediatricians and family physicians in terms of age and gender (p > 0.05). Nearly half of all physicians (49%) stated that they do not have sufficient knowledge about OVs. Pediatricians (64%) stated that they have sufficient knowledge at a higher rate than family physicians (37%) (p = 0.000). Physicians who declared having sufficient knowledge informed families about OVs more frequently than those with insufficient knowledge (p = 0.000). Pediatricians provide information about OVs more frequently than family physicians (p = 0.001). Rotavirus and meningococcal vaccines were the most frequently recommended vaccines.

Conclusions: Rotavirus and meningococcal B were the most recommended OVs. About half of the physicians participating in the study stated that they did not have sufficient knowledge about OVs. Physicians with sufficient knowledge of OVs recommend OVs more frequently.

## Introduction

Immunization is a leading factor affecting pediatric mortality and morbidity in public health [1-3]. Healthcare professionals informing families about vaccinations affect vaccination rates [1]. Many vaccines in Turkey are in routine vaccination programs and are provided unpaid. In addition, optional vaccines (OVs), such as human papillomavirus, meningococcus, influenza, and rotavirus, are also available. The recommendation of OVs is recognized as an important part of health care [4,5]. The coverage of OVs may differ in different populations for various reasons, including low awareness and financial costs [6]. Due to Turkey's geographical proximity to warring countries with low vaccination coverage, children living in regions with frequent immigration from these countries have an increased likelihood of vaccine-preventable diseases [7]. Routine childhood vaccinations decreased by 2-5% during the COVID-19 pandemic in Turkey [8]. In addition to these epidemiological risks, parental negative perceptions, attitudes, and lack of knowledge about vaccination may gradually reduce the high vaccination rates achieved so far. In different populations, inadequate vaccination is associated with inadequate vaccination services and inadequate parental knowledge, attitudes, and concerns [9,10]. Vaccination interventions should be targeted according to concerns such as parents' knowledge, attitudes, beliefs, and vaccine hesitancy to maintain high rates and a positive family attitude [11].

This study aims to evaluate the current knowledge and behaviors of healthcare professionals (family physicians/pediatricians) regarding optional childhood vaccines and vaccinations. The fact that the study was carried out during the pandemic period reveals the attitudes toward recommending OVs despite the pandemic in the region.

## Materials and methods

This research was conducted between June 2020 and April 2021 using a questionnaire for pediatricians and family physicians working in Manisa province, Turkey. This study was approved by the Institutional Review Board of Manisa Celal Bayar University (20.478.486/June 2020). This study aimed to investigate the knowledge and attitudes of family physicians and pediatricians regarding OVs, which are not included in the national childhood vaccination program.

Inclusion criteria were to be a pediatrician's assistant/specialist or to be a family physician/family physician's assistant/specialist, and to be active in the field of child health. Participants who did not give written consent were excluded.

The vaccines included in the Extended Immunization Program of Turkey (2020) are tuberculosis, hepatitis B, hepatitis A, polio, diphtheria, tetanus, acellular pertussis, *Haemophilus influenzae* type B, conjugated pneumococcus, and measles-mumps-rubella. These vaccines are administered free of charge to all children. OVs are rotavirus, influenza, human papillomavirus (HPV), tetravalent meningococcal (A, C, Y, W), and B type meningococcal vaccines and are paid by families or private health insurance.

A questionnaire about optional and routine vaccines was prepared based on the current literature [1,12]. Demographic characteristics, time of graduation from medical school, duration of work in pediatrics and family medicine, and attitudes toward OVs recommendation were questioned (to whom OVs are recommended (every child, those with risk factors, other, none), if not, the reason (high cost, not safe, not necessary, insufficient knowledge, other)). Questions on optional vaccination information adequacy status, routine information to families (yes/no), and healthcare workers who provided the information (physician, vaccination nurse, patient’s physician) were asked. Physicians were also asked about the optional vaccinations of their children. The questionnaire was pre-tested with five family physicians and five pediatricians. The participants were interviewed face-to-face and by the internet/phone after revision.

The sample size was calculated using power analysis. When the power was 85% (α = 0.05, d (effect size) = 0.40), it was calculated that there would be a minimum of 113 people in each group. Physicians were included in the study with an improbable sample without using any sampling method, and voluntarily by telephone or face-to-face interview method.

All statistical analyses were performed using SPSS 20.0 package program (IBM Corp., Armonk, NY). Continuous variables obtained by measurement were expressed as mean ± standard deviation; categorical variables in sociodemographic, clinical data, and scales were expressed as percentages and numbers. The Student's t-test was used in independent groups and the nonparametric Mann-Whitney U test was used in the comparison of the mean between two groups with normal distribution among numerical variables. Chi-square analysis and Fisher's exact test were used to compare categorical data. A p-value of <0.05 was considered statistically significant in all analyses.

## Results

A total of 241 physicians participated in our study (317 physicians were reached out, and the rate of participation and filling out the form was 76%). Questionnaires with insufficient data were excluded (n = 14). A total of 227 participants, including 115 pediatricians and 112 family physicians, were included in the study.

The mean age of pediatricians and family physicians was 33.42 ± 8.25 years and 35.46 ± 11.09 years, respectively. There was no significant difference between pediatricians and family physicians in terms of age and gender (p > 0.05) (Table 1).

**Table 1:** Sociodemographic characteristics of pediatricians and family physicians * Other: referral to the vaccination nurse/referral to the patient’s own pediatrician or family physician.

	Pediatricians (n = 115) (%)	Family physicians (n = 112) (%)	P	Physicians with children (n = 99) (%)	Physicians without children (n = 128) (%)	P
Gender, male/female	46/69	46/66	0.869	43/56	49/79	0.433
Age (years)	33.42 ± 8.25	35.46 ± 11.09	0.998	41.62 ± 10.26	28.87 ± 4.22	0.000
Years in practice						
0-5 years	47 (40%)	59 (52%)	0.001	11 (11%)	95 (75%)	0.000
6-10 years	36 (31%)	14 (12%)		21 (21%)	29 (23%)	
11-15 years	16 (13%)	10 (8%)		24 (24%)	2 (1%)	
>16 years	16 (13%)	29 (25%)		43 (44%)	2 (1%)	
Workplace						
University hospital	86 (74%)	53 (47%)	0.000	38 (38%)	101 (79%)	0.000
Public hospital	22 (19%)	2 (2%)		13 (13%)	10 (8%)	
Private office/clinic	8 (7%)	0		8 (8%)	0	
Primary healthcare center	0	57 (51%)		40 (41%)	17 (13%)	
Town center	94 (82%)	96 (86%)	0.371	78 (79%)	112 (88%)	0.143
Commuter town	21 (18%)	16 (14%)		21 (21%)	16 (12%)	
Income and expense status						
Income more than expenses	52 (45%)	45 (40%)	0.734	38 (38%)	59 (46%)	0.441
Income equals expense	57 (51%)	60 (54%)		54 (55%)	63 (49%)	
Income missing from expenses	6 (5%)	7 (6%)		7 (7%)	6 (5%)	
Knowledge about optional vaccines						
Sufficient	74 (64%)	42 (37%)	0.000	66 (67%)	50 (39%)	0.143
Insufficient	41 (36%)	70 (63%)		33 (33%)	78 (61%)	
Informing families about optional vaccinations						
Anytime	44 (38%)	47 (%42)	0.563	52 (%53)	39 (%30)	0.005
If the family requests information	49 (42%)	40 (36%)		34 (34%)	55 (43%)	
Sometimes/rarely	16 (14%)	21 (19%)		10 (10%)	27 (21%)	
Never	6 6%)	4 (3%)		3 (3%)	7 (6%)	
Optional vaccination information	n = 115	n = 112				
Physician	80 (69%)	53 (47%)	0.001	70 (71%)	63 (49%)	0.005
Other*	35 (31%)	59 (53%)		29 (29%)	65 (51%)	

Optional vaccine recommendations by all physicians

Rotavirus and meningococcal B vaccines were the most recommended vaccines (96.91%). Tetravalent meningococcal, influenza, and HPV vaccines were recommended by 96.47%, 92.95%, and 86.78%, respectively. There was no statistically significant difference in the recommendation of any OVs in terms of age, gender, years in practice, and graduation date of all physicians (p > 0.05).

The frequency of information was stated as anytime (40.1%), upon request of the family (39.2%), sometimes or rarely (16.3%), and never (4.4%). When questioned about the reasons for the lack of information, 51.8% of participants stated the high number of outpatients, 24.1% forgot to inform about OVs, and 7.3% stated that the coverage of the routine vaccination schedule was sufficient.

Nearly half of all physicians (49%) stated that they do not have sufficient knowledge about OVs. Pediatricians (64%) stated that they have sufficient knowledge at a higher rate than family physicians (37%) (p = 0.000). Physicians who declared having sufficient knowledge informed families more frequently than those with insufficient knowledge (p = 0.000).

Optional vaccination recommendations by pediatricians and family physicians

Information about OVs was provided more frequently by pediatricians (69%) than family physicians (47%) (p = 0.001). Family physicians also refer families to the immunization nurse and the child's primary follow-up physician for information.

HPV vaccine was recommended more frequently by family physicians (70%) than by pediatricians (53%) to be included in the extended immunization program (p = 0.010) (Table 2).

**Table 2:** Distribution of optional vaccines recommended to be included in the national extended immunization program HPV: human papillomavirus vaccine.

Vaccines	Pediatricians (n = 115) (%)	Family physicians (n = 112) (%)	Total (n = 227) (%)	P	Physicians with children (n = 99) (%)	Physicians without children (n = 128) (%)	Total (n = 227) (%)	P
Influenza	41 (35%)	38 (33%)	79 (34%)	0.785	37 (37%)	42 (33%)	79 (35%)	0.474
Meningococcus ACWY	98 (85%)	88 (78%)	186 (81%)	0.193	85 (86%)	101 (79%)	186 (82%)	0.177
Meningococcus B	99 (86%)	92 (82%)	191 (84%)	0.416	86 (87%)	105 (82%)	191 (84%)	0.322
Rotavirus	92 (80%)	98 (87%)	190 (83%)	0.126	81 (82%)	109 (85%)	190 (83%)	0.500
HPV	62 (53%)	79 (70%)	141 (62%)	0.010	64 (65%)	77 (60%)	141 (62%)	0.489

There was no significant difference in the OV recommendation rates of pediatricians and family physicians (Table 3).

**Table 3:** Optional vaccination recommendations by pediatricians/family physicians or physicians with and without children HPV: human papillomavirus vaccine.

	Pediatricians (n = 115) (%)	Family physicians (n = 112) (%)	P	Physicians with children (n = 99) (%)	Physicians without children (n = 128) (%)	P
Influenza	107 (93%)	104 (93%)	0.956	96 (97%)	115 (89%)	0.038
Meningococcus A	113 (98%)	106 (94%)	0.167	92 (93%)	127 (99%)	0.023
Meningococcus B	113 (98%)	107 (95%)	0.440	93 (94%)	127 (99%)	0.088
Rotavirus	110 (95%)	110 (98%)	0.446	93 (94%)	127 (99%)	0.045
HPV	113 (98%)	106 (94%)	0.167	87 (88%)	110 (86%)	0.668

There was no difference between the patient groups for which they were recommended (Table 4).

**Table 4:** Distribution of pediatricians and family physicians according to patient groups for which optional vaccines were recommended

	Pediatricians (n = 115) (%)	Family physicians (n = 112) (%)	P	Physicians with children (n = 99) (%)	Physicians without children (n = 128) (%)
Influenza						
All children	33 (29%)	40 (36%)	0.713	31 (31%)	42 (33%)
Those with risk factors	73 (63%)	63 (56%)		63 (64%)	73 (57%)
Any child	8 (7%)	8 (7%)		3 (3%)	13 (10%)
Other	1 (1%)	1 (1%)		2 (2%)	0
Meningococcus ACWY						
All children	82 (71%)	71 (64%)	0.391	74 (75%)	79 (61%)
Those with risk factors	30 (26%)	34 (30%)		17 (17%)	47 (37%)
Any child	2 (2%)	6 (5%)		7 (7%)	1 (1%)
Other	1 (1%)	1 (1%)		1 (1%)	1 (1%)
Meningococcus B						
All children	86 (75%)	73 (64%)	0.326	77 (78%)	82 (64%)
Those with risk factors	26 (22%)	32 (30%)		15 (15%)	43 (34%)
Any child	2 (2%)	4 (3%)		5 (5%)	1 (1%)
Other	1 (1%)	3 (3%)		2 (2%)	2 (1%)
Rotavirus						
All children	93 (81%)	91 (81%)	0.519	78 (79%)	106 (83%)
Those with risk factors	16 (14%)	16 (14%)		12 (12%)	20 (15%)
Any child	5 (4%)	2 (2%)		6 (6%)	1 (1%)
Other	1 (1%)	3 (3%)		3 (3%)	1 (1%)
Human papillomavirus						
All children	69 (60%)	68 (61%)	0.510	57 (58%)	80 (63%)
Those with risk factors	30 (26%)	23 (21%)		26 (26%)	27 (21%)
Any child	14 (12%)	16 (14%)		12 (12%)	18 (14%)
Other	2 (2%)	5 (4%)		4 (4%)	3 (2%)

The meningococcal B vaccine was under-recommended by pediatricians due to its high cost (p = 0.041). The HPV vaccine was also under-recommended by family physicians due to its high cost (p = 0.021) (Table 5).

**Table 5:** Reasons why pediatricians and family physicians do not recommend optional vaccines to all children

	Pediatricians	Family physicians	P	Physicians with children	Physicians without children	P
Influenza	(n = 82)	(n = 72)		(n = 68)	(n = 86)	
Some children should be vaccinated	51	48	0.563	43	56	0.809
High cost	7	6	0.964	3	10	0.110
Must be repeated annually	19	21	0.397	17	23	0.806
Not safe	1	0	1.000	1	0	0.442
Not required	9	14	0.141	11	12	0.701
Not effective	9	4	0.227	5	8	0.666
Insufficient information	3	4	0.573	2	5	0.465
Meningococcus ACWY	(n = 33)	(n = 41)		(n = 25)	(n = 49)	
Some children should be vaccinated	21	22	0.387	13	30	0.447
High cost	9	8	0.430	5	11	0.881
Not safe	2	1	0.583	2	1	0.262
Not required	1	1	1.000	2	0	0.111
Insufficient information	5	13	0.099	4	14	0.233
Meningococcus B	(n = 30)	(n = 38)		(n = 21)	(n = 47)	
Some children should be vaccinated	15	18	0.916	6	27	0.047
High cost	13	8	0.041	10	11	0.064
Not safe	1	1	1.000	2	0	0.098
Not required	1	1	1.000	2	0	0.098
Insufficient information	5	14	0.076	5	14	0.541
Rotavirus	(n = 22)	(n = 21)		(n = 21)	(n = 22)	
Some children should be vaccinated	9	12	0.287	11	10	0.650
High cost	4	8	0.146	6	6	0.924
Not safe	3	0	0.233	1	2	1.000
Not required	4	2	0.664	5	1	0.095
Insufficient information	5	3	0.698	2	6	0.240
Human papillomavirus	(n = 46)	(n = 44)		(n = 42)	(n = 48)	
Some children should be vaccinated	20	19	0.977	22	17	0.105
High cost	7	16	0.021	10	13	0.722
Not safe	2	4	0.429	3	3	1.000
Not required	11	8	0.505	4	15	0.012
Insufficient information	9	9	0.916	9	9	0.751

Optional vaccination recommendation attitudes of physicians with and without children

The mean age of physicians with children (n = 99; 41.62 ± 10.26 years) was higher than physicians without children (n = 128; 28.87 ± 4.22 years) (p = 0.000). No statistically significant difference was found in terms of gender with and without children (p > 0.05) (Table 1). Physicians without children had shorter years in practice than those with children and worked more frequently in university hospitals (p = 0.000 and p = 0.000, respectively). The most missing vaccines of the physician’s children were HPV (n = 34), meningococcal B (n = 34), tetravalent meningococcus (n = 28), influenza (n = 23), and rotavirus (n = 21).

While the influenza vaccine is recommended more frequently by physicians with children (p = 0.038), tetravalent meningococcal (p = 0.023) and rotavirus vaccines (p = 0.045) are recommended more frequently by physicians who do not have children (Table 3). It was found that physicians who do not have children recommend tetravalent meningococcal and meningococcal B vaccines more frequently to children with risk factors than physicians who have children (p = 0.002 and p = 0.005, respectively) (Table 4).

There was no significant difference in the vaccination preferences for their children between physicians (n = 48) and pediatricians (n = 51 (p > 0.05).

When family physicians and pediatricians were compared about the vaccination status of their children, the HPV vaccine was reported to be missing in a higher number in the family physicians group (n = 22 vs. 12; p = 0.020). No significant difference was found between the two groups in other OVs (p > 0.05). Only 22 pediatricians and 10 family physicians stated that no OV was missing in their children (p = 0.018).

## Discussion

Studies conducted during the COVID-19 pandemic period have shown a decrease in childhood vaccine coverage and the total number of vaccines administered, and an increase in missed vaccines [13]. In this study, we have reported the current attitudes of physicians in our province regarding pediatric OVs during the pandemic period. Strikingly, 49% of all physicians in our study reported insufficient information on OVs. The number of family physicians who stated that their knowledge about OVs was insufficient was higher than pediatricians. Physicians without children reported their knowledge as insufficient than those with children. Physicians who think that OVs' knowledge is sufficient often informed families themselves. Rotavirus and meningococcal B vaccines were the most recommended vaccines. The least recommended vaccine was influenza, but it was recommended more frequently for children with risk factors.

The most recommended vaccine to be included in the national immunization program was meningococcal B, with the influenza vaccine being the least recommended. Family physicians were more likely to recommend HPV to be included in the immunization program than pediatricians.

The rotavirus vaccine was the most preferred vaccine by physicians for their children. Physicians without children recommended tetravalent meningococcal, meningococcal B, and rotavirus vaccines more than physicians who had children.

Physicians providing information about OVs increase families' knowledge of the subject [14]. Families informed by healthcare professionals have more positive attitudes about vaccines, their safety, effectiveness, and risks. They evaluate the severity and risk of the potential disease for the child [14,15]. Families informed by healthcare professionals are more likely to vaccinate their children [16,17]. The number of studies conducted in Turkey reflecting the knowledge of physicians about OVs is limited. Similar to some other studies in Turkey, 49% of all participants report that their knowledge is insufficient [12,18]. Different rates were also reported on the subject. In a study by Elitok et al. [12], adequate knowledge rates of pediatricians and family physicians regarding adolescent OVs were reported as 10% and 5.4%, respectively, with significant statistical differences. In our results, pediatricians (64%) stated that they had more sufficient information than family physicians (37%). On the other hand, physicians who stated that they had sufficient knowledge in our study often informed families about the vaccine themselves. Family physicians often refer to the vaccination nurse and the child's primary physician for vaccination information. In a small sample size study (n = 78), 23.1% of family physicians reported sufficient knowledge, and all of them were among those who received training on OVs [18]. The beliefs of healthcare professionals have been shown to influence their attitudes toward vaccination [19]. In a systematic review by Leung et al. [20], vaccination rates increased after training on HPV vaccination. In another study by Suryadevara et al. [21], the vaccination rates of pediatricians and nurses increased up to 20% after HPV vaccination training. With training and national guidelines for physicians in our country, optional vaccination and the knowledge level of healthcare providers may increase.

During the pandemic period, 99.1% of physicians recommend at least one OV to their patients. Similarly, in the pre-pandemic period, Parlakay et al. [22] reported that all pediatricians participating in the study recommended at least one OV. In the same study, the meningococcal vaccine was recommended by all pediatricians, the rotavirus was recommended by 82.8%, and the HPV vaccine was recommended by 89.2% [22]. In a small sample size study evaluating only family physicians, rotavirus was the most recommended vaccine (83.8%), followed by influenza (62.2%), meningococcal vaccine (39.1%), and HPV (27.5%) [18]. Çataklı et al. [23] found that pediatricians recommend tetravalent meningococcal and rotavirus vaccines significantly more frequently than family physicians in a province of Turkey (Ankara). In a study covering all regions of Turkey, the tetravalent meningococcal vaccine was recommended by 88.6%, the HPV vaccine by 86.5%, and the influenza vaccine was recommended by 91.8% of pediatricians, while the tetravalent meningococcal vaccine was recommended by 79.3%, HPV vaccine by 72.9%, and influenza vaccine by 89.6% of family physicians. A statistically significant difference was found between the recommendations of family physicians and pediatricians for all OVs [12]. In our study, optional vaccination recommendations of family physicians and pediatricians were high, and no significant difference was found between the two groups. The lack of difference may be related to the fact that it was made for all pediatric age groups and covers a limited area. Similar to other studies, rotavirus and meningococcal B were the most recommended OVs for children [18,22,23].

The years in practice in the profession affect vaccination recommendations. Young physicians recommend OVs more often [24-27]. We found no significant difference between graduation date/time of employment and optional vaccination recommendations. However, physicians who do not have children were younger and recommended more OVs than those who had children. The fact that most childless physicians work in an academic setting may explain the high recommendation rates for vaccination [28].

Hesitant attitudes are decreasing among healthcare workers with the national immunization programs [29]. Physicians recommend vaccines included in the national immunization program and funded by the state more frequently [29,30]. In this way, the knowledge of the families about the vaccines and the vaccination rates increased, the anxiety in the family about the vaccination and vaccine-related diseases decreased, and healthcare professionals informed the families about the necessity of the vaccines more frequently [31]. In Turkey, family physicians and pediatricians stated that rotavirus (48.5%), tetravalent meningococcal (44.8%), and HPV (36.7%) vaccines should be included in the national extended immunization program. Abstaining physicians (rotavirus (18.1%), tetravalent meningococcus (30.1%), and HPV (31.1%)) were also reported in the same study [23]. These rates are lower than our rates, and the reason for this difference can be explained by the inclusion of abstaining physicians in this number or regional differences. In a study by Dinleyici et al. [32], 74.3% of 339 physicians stated that meningococcal vaccines should be included in the national immunization program. Celep [18] found these rates to be 55.1% for rotavirus, 59% for meningococcus, 52.6% for HPV, and 32.1% for influenza in a study conducted among family physicians. Meningococcal vaccines were found to be in the first place to be included in the national extended immunization scheme, followed by HPV and rotavirus [22]. In this study, higher rates were found for the inclusion of OVs in the national immunization scheme compared to the literature in Turkey, which is similar to our data. Both studies were conducted at similar times. According to other reports, this difference may be related to increased awareness of OVs over the years. Differently, the HPV vaccine was recommended by 70% of family physicians, and they stated that it should be included in the national immunization program at a significantly higher rate than pediatricians. In addition, family physicians state that they do not recommend the HPV vaccine to all children because of its high cost compared to pediatricians. For this reason, family physicians may have thought that the HPV vaccine should be included in the national immunization program, considering the cost of the HPV vaccine and that people should be able to access this vaccine free of charge. Family physicians also take part in primary cervical cancer screening of women in Turkey and they work in “cancer early diagnosis, screening, and education centers” (KETEM) [33,34]. This may affect the HPV vaccine recommendation attitude of family physicians. One of our remarkable findings was that female physicians were not different from males in their HPV recommendations.

Physicians' attitudes toward vaccines of their children reflect their beliefs and affect large-scale vaccination [1,35]. Of the pediatricians and family physicians, 35.2% administered the rotavirus vaccine, 26.7% administered the tetravalent meningococcal vaccine, and 13.2% administered the HPV vaccine to their children in Turkey [23]. In a study conducted in Switzerland, 12.8% and 14.3% of pediatricians and family physicians, respectively, stated that they recommend influenza vaccines to their children and no difference was found between family physicians and pediatricians [1]. In our study, the most preferred OVs by physicians with children were meningococcal B, tetravalent meningococcal, rotavirus, HPV, and influenza vaccines, respectively. In a study conducted in Turkey, similar to our data, it has been found that although rotavirus is more common, meningococcal vaccination is also at the forefront [23]. Physicians without children were working in university hospitals and were younger in their profession and age. Physicians who do not have children stated that they recommend OVs more frequently than those who have children, except for meningococcal B and HPV vaccines. The recommendation of meningococcal ACWY, meningococcal B, and rotavirus vaccines by younger physicians among both pediatric and family physicians also makes our findings consistent [24,25]. In addition, physicians who did not have children in our study stated that their knowledge was insufficient. This may be related to the information resources of young physicians about vaccines. It has been reported that young physicians follow information about vaccines from the internet more than guidelines [36].

We found the rotavirus and meningococcal B vaccines to be the most frequently recommended vaccines. In the United States, it was stated that the meningococcal B vaccine was recommended in routine pediatric visits with a frequency of 51% and 31% among pediatricians and family physicians, respectively, and in another study conducted in Italy, it was recommended by pediatricians with a frequency of 93% [37,38]. In a study evaluating the meningococcal vaccine recommendations of pediatricians in Turkey, 40.7% stated that they recommend it to their patients [32]. The rate of recommendation of conjugated meningococcal vaccine to patients by Dinleyici et al. [32] was reported as 53.6% among pediatricians and adult physicians. In a study by Parlakay et al. [22] conducted in 2020, all participating pediatricians recommended the meningococcal vaccine. In our study, 71.3% and 63.4% of pediatricians and family physicians recommend the meningococcal ACWY vaccine, and again, 74.7% and 64.28% of pediatricians and family physicians recommend the meningococcal B vaccine to all their patients, respectively. In addition, it was noted that meningococcal ACWY and meningococcal B vaccines were recommended by physicians who did not have children to children with higher risk factors. Ferrara et al. [37] discussed the high recommendation rate of 93% in their study, and it was found that this rate decreased to 35.5%, especially when parameters such as informing families and controlling the vaccination schedule were also evaluated. Our rates are similarly high, but there is a need to compare families with vaccination rates.

Rotavirus infections and viral gastroenteritis cases continue to be important problems in Turkey [39]. This may be the reason why the rotavirus vaccine is the leading recommended OV in studies conducted in Turkey. It has been determined that 38.7% of pediatricians in Turkey still follow up on more than 10 patients with rotavirus gastroenteritis per year [22]. In the study of Parlakay et al. [22], 89.2% of the participating pediatricians recommended the rotavirus vaccine, and it is in second place after the meningococcal vaccine, which supports our findings and is similar for family physicians and pediatricians. However, although the rotavirus vaccine is frequently recommended, it is still not at the expected rate in Turkey [4,40]. In our study, it was stated that the rotavirus vaccine should be included in the national immunization program by 92.43% of the physicians. Lack of government funding may also be related to low vaccination rates despite the high recommendation rate.

While there is no difference between the recommendations of family physicians and pediatricians for HPV vaccination in Turkey [41], similarly, no difference was found between family physicians and pediatricians in our findings.

The American Academy of Pediatrics (APA) recommends annual flu vaccination for all children aged ≥ six months [42]. Vaccination of anyone older than six months provides herd immunity and has the potential to reduce flu illness, flu-related complications, and flu-related school or job loss [42,43]. Vaccination of household contacts and caregivers of oncology patients receiving chemotherapy who are unable to produce an adequate immune response to the vaccine remains the primary means of reducing the risk of influenza [44]. Influenza vaccines provide significant protection against influenza-related hospitalization in the pediatric population, and annual vaccination is important [45]. The lack of recommendation for influenza vaccines to families by healthcare professionals is an important reason for not being vaccinated. The major barrier to influenza vaccination in children hospitalized for acute respiratory illness was the lack of opportunities for parents to access vaccination services and seek advice from a healthcare provider [46]. It was found that there was an 8.8% influenza vaccination rate in a tertiary hospital in Ankara. Influenza vaccination was recommended to 72% of vaccinated children by their physicians [47]. In a study conducted in Turkey, none of the 131 pediatric patients who were hospitalized for influenza lower respiratory tract infections between 2016 and 2018 (60.3% without comorbidity) were vaccinated [44]. In a study conducted among healthcare workers in a third-line pediatric hospital in Turkey, the rate of vaccination among healthcare workers was reported as 14.8% in the winter of 2016-2017, while the rate of those who were vaccinated at least once in their lives was 60.2% [48]. Despite the recommendation rates of over 90% in our study, the difference in vaccination rates of both healthcare workers and children reported from Turkey underlines the need for more studies to examine the reasons in this area.

Limitations

This study was conducted to determine the behaviors of pediatricians and family physicians regarding OVs. However, being in a limited region and short duration caused the sample to consist of a small group. In our study, the knowledge level of physicians about vaccines was not measured, and the adequacy of knowledge was evaluated according to their statements. In addition, information resources and training on OVs and vaccines in the routine national vaccination calendar were not questioned. How often physicians see the diseases that can be prevented by OVs included in our study in their routine work life, their level of knowledge about these diseases, and how often they prescribe these vaccines were not among the questions we asked. In our study, while physicians were asked to state the reasons for not recommending OVs, they were not asked to state the reasons for recommending these vaccines. It was not questioned whether the OV was administered in the centers where the physicians worked or by whom the OV was administered in the center. Whether family physicians screen for cervical cancer in their units is not among the contents of our study. Another limitation of our study is that we did not differentiate between adolescent and childhood periods.

## Conclusions

OV recommendation rates were found to be quite high in this study. Rotavirus and meningococcal B were the most recommended OVs. About half of all physicians evaluated their knowledge about OVs as insufficient. Especially family physicians and physicians who do not have children stated that their knowledge was insufficient. There was no difference in the recommendation of OVs between pediatricians and family physicians.
